# High levels of imported asymptomatic malaria but limited local transmission in KwaZulu-Natal, a South African malaria-endemic province nearing malaria elimination

**DOI:** 10.1186/s12936-020-03227-3

**Published:** 2020-04-15

**Authors:** Jaishree Raman, Laura Gast, Ryleen Balawanth, Sofonias Tessema, Basil Brooke, Rajendra Maharaj, Givemore Munhenga, Power Tshikae, Vishan Lakan, Tshiama Mwamba, Hazel Makowa, Lindi Sangweni, Moses Mkhabela, Nompumelelo Zondo, Ernest Mohulatsi, Zuziwe Nyawo, Sifiso Ngxongo, Sipho Msimang, Nicole Dagata, Bryan Greenhouse, Lyn-Marie Birkholtz, George Shirreff, Rebecca Graffy, Bheki Qwabe, Devanand Moonasar

**Affiliations:** 1 Centre for Emerging Zoonotic and Parasitic Diseases, National Institute for Communicable Diseases, a Division of the National Health Laboratory Service, Johannesburg, Gauteng South Africa; 2 Wits Research Institute for Malaria, Faculty of Health Sciences, University of Witwatersrand, Johannesburg, Gauteng South Africa; 3 UP Institute for Sustainable Malaria Control, Faculty of Health Sciences, University of Pretoria, Pretoria, Gauteng South Africa; 4 Clinton Health Access Initiative, Pretoria, Gauteng South Africa; 5 Department of Medicine, University of California-San Francisco, San Francisco, USA; 6 Office of Malaria Research, South African Medical Research Council, Durban, KwaZulu-Natal South Africa; 7 Department of Biochemistry, Genetics and Microbiology, University of Pretoria, Pretoria, Gauteng South Africa; 8 KwaZulu-Natal Provincial Malaria Control Programme, Jozini, KwaZulu-Natal South Africa; 9 Humana People to People, Jozini, KwaZulu-Natal South Africa; 10 KwaZulu-Natal Provincial Department of Health, Pietermaritzburg, KwaZulu-Natal South Africa; 11 Malaria Vector Borne and Zoonotic Diseases, National Department of Health, Pretoria, Gauteng South Africa

**Keywords:** Malaria, South Africa, KwaZulu-Natal, Residual transmission, Asymptomatic carriage, Elimination, Vector control, Rapid diagnostic tests, Malaria importation, KAP

## Abstract

**Background:**

KwaZulu-Natal, one of South Africa’s three malaria endemic provinces, is nearing malaria elimination, reporting fewer than 100 locally-acquired cases annually since 2010. Despite sustained implementation of essential interventions, including annual indoor residual spraying, prompt case detection using malaria rapid diagnostics tests and treatment with effective artemisinin-based combination therapy, low-level focal transmission persists in the province. This malaria prevalence and entomological survey was therefore undertaken to identify the drivers of this residual transmission.

**Methods:**

Malaria prevalence as well as malaria knowledge, attitudes and practices among community members and mobile migrant populations within uMkhanyakude district, KwaZulu-Natal were assessed during a community-based malaria prevalence survey. All consenting participants were tested for malaria by both conventional and highly-sensitive falciparum-specific rapid diagnostic tests. Finger-prick filter-paper blood spots were also collected from all participants for downstream parasite genotyping analysis. Entomological investigations were conducted around the surveyed households, with potential breeding sites geolocated and larvae collected for species identification and insecticide susceptibility testing. A random selection of households were assessed for indoor residual spray quality by cone bioassay.

**Results:**

A low malaria prevalence was confirmed in the study area, with only 2% (67/2979) of the participants found to be malaria positive by both conventional and highly-sensitive falciparum-specific rapid diagnostic tests. Malaria prevalence however differed markedly between the border market and community (p < 0001), with the majority of the detected malaria carriers (65/67) identified as asymptomatic Mozambican nationals transiting through the informal border market from Mozambique to economic hubs within South Africa. Genomic analysis of the malaria isolates revealed a high degree of heterozygosity and limited genetic relatedness between the isolates supporting the hypothesis of limited local malaria transmission within the province. New potential vector breeding sites, potential vector populations with reduced insecticide susceptibility and areas with sub-optimal vector intervention coverage were identified during the entomological investigations.

**Conclusion:**

If KwaZulu-Natal is to successfully halt local malaria transmission and prevent the re-introduction of malaria, greater efforts need to be placed on detecting and treating malaria carriers at both formal and informal border crossings with transmission blocking anti-malarials, while ensuring optimal coverage of vector control interventions is achieved.

## Background

**Fig. 1:**
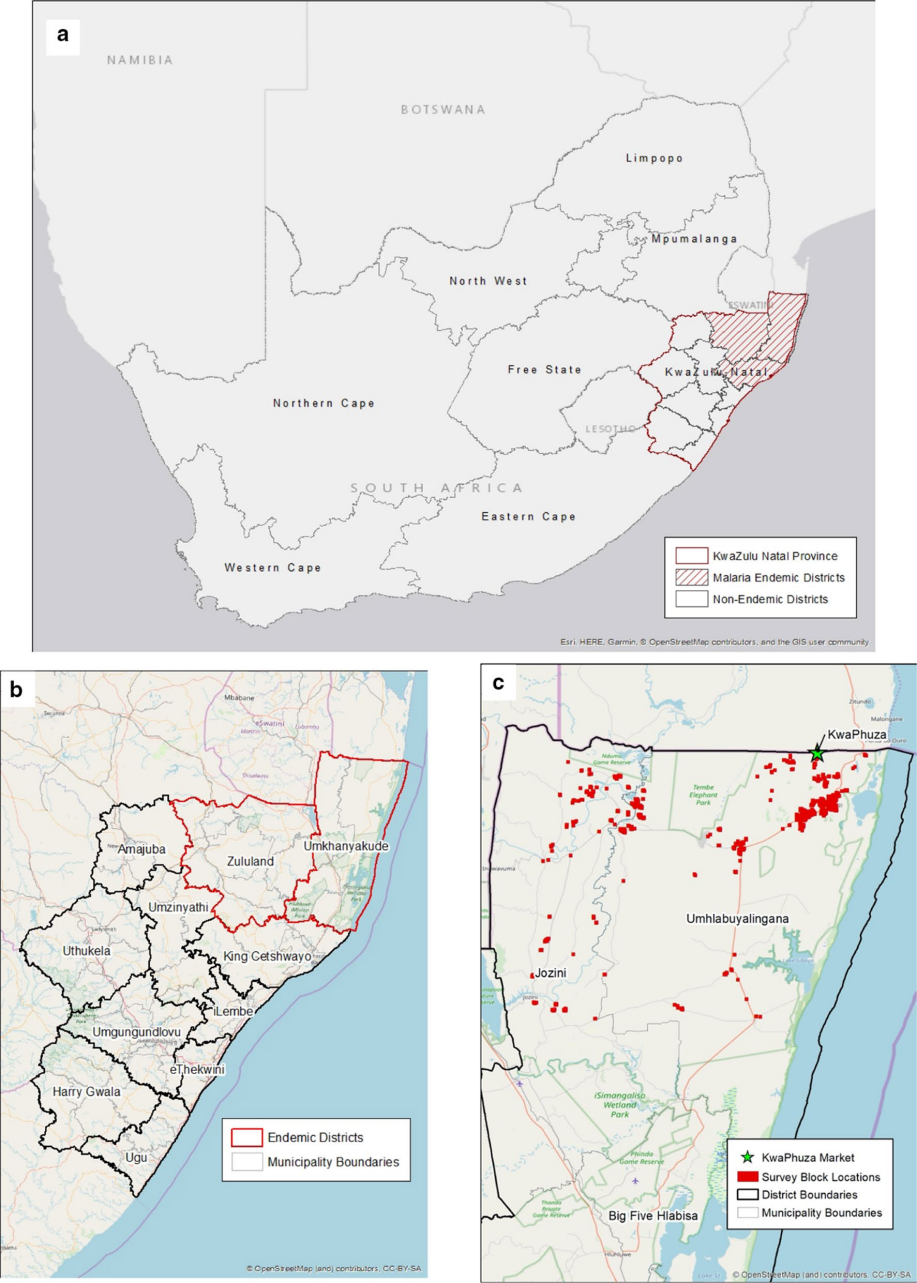
**a** Malaria endemic and non-endemic provinces in South Africa; **b** the endemic and non-endemic districts of KwaZulu-Natal and **c** the localities sampled within the community and by the mobile surveillance units. Green star denotes KwaPhuza border market

With a sustained national malaria incidence of less than 1 malaria case per 1000 population at risk, South Africa began implementing its 5-year malaria elimination strategy [1] in 2012. Achieving the country’s elimination target of 2018 proved challenging due to financial and logistical constraints, resulting in suboptimal coverage of key interventions and an upsurge in cases [2]. Using the World Health Organization’s Global Technical Strategy for Malaria 2016–2030 [3] as a guide, South Africa revised its malaria elimination strategy to include a phased district-level approach to malaria elimination [4]. As part of this phased approach, all three malaria-endemic districts within KwaZulu-Natal (KZN; Fig. 1a), one of South Africa’s three malaria-endemic provinces, are being targeted for sub-national malaria elimination verification by 2021.

KwaZulu-Natal bore the brunt of the 1999/2000 malaria epidemic, reporting in excess of 40 000 cases and over 300 deaths in 2000 [5]. Drivers of the outbreak included the establishment of sulfadoxine-pyrimethamine resistant *Plasmodium falciparum* parasites [6, 7] and the emergence of pyrethroid-resistant *Anopheles funestus* vector populations [8]. Responses to the outbreak included the successful implementation of an artemisinin-based combination therapy treatment policy, a first in Africa [9], and the re-introduction of dichlorodiphenyltrichloroethane (DDT)-based indoor residual spraying [10] in the province. Sustained implementation of these and other strategic interventions, including routine active case detection and a robust cross-border malaria collaboration with southern Mozambique and Eswatini [11], have substantially reduced reported case numbers and local transmission in KZN.

Since 2005, KZN has accounted for less than one percent of South Africa’s malaria burden, with its three endemic districts, uMkhanyakude, King Cetshwayo and Zululand (Fig. 1b), achieving and maintaining very low transmission intensity status [12] since 2010 [5]. This, despite the regional upsurges in malaria cases in 2014 [13], and most recently in 2017 [2], when South Africa reported over 30,000 cases, the highest number of cases since the 1999/2000 epidemic. While the current control strategies appear to provide adequate protection against upsurges, they have not stemmed persistent residual local transmission, particularly in uMkhanyakude district, KZN.

It is against this backdrop that this study was undertaken to identify, describe and define the factors driving and sustaining low-level malaria transmission in KZN. It is envisaged that the data generated will contribute to a holistic understanding of the various elements contributing to residual transmission in northern KZN, and inform elimination intervention selection and implementation towards sub-national malaria elimination by 2021.

## Methods

### Study site

This study was conducted in areas identified as hotspots of local transmission, namely the municipalities of Jozini (a peri-urban area) and uMhlabuyalingana (rural, border area) in uMkhanyakude district, KZN, over a 6-week period in February and March 2018. Malaria transmission in this region occurs mainly during the hot, wet summer months from September to May, with *P. falciparum* and *Anopheles arabiensis* the dominant parasite [14] and vector [15] species, respectively. Annual insecticide-based indoor residual spraying (IRS) of households in communities with reported locally-acquired cases is the core vector control intervention in this area. Spray operations generally take place at the beginning of the malaria season, between September and November. Standard malaria case management interventions included diagnosis using the First Response^®^ falciparum-specific rapid diagnositic test (RDT; First Response™ Malaria Ag *P. falciparum* HRP2 Detection Rapid Card Test, Premier Medical Corporation Ltd, India) or blood smears and treatment with the artemisinin-based combination therapy, artemether–lumefantrine (Coartem^®^, Novartis Pharma, South Africa).

### Study design

A mixed-methods approach was employed to facilitate an in-depth examination of potential malaria risk factors. The first approach was a community-based, household-level malaria prevalence survey in which participants were tested for malaria using standard and highly-sensitive falciparum-specific RDTs, and were assessed on their malaria-related knowledge, attitudes and practices (KAP). To understand the movements of the migrant and mobile populations (MMPs) and their potential contribution to sustained transmission, the second component comprised an assessment of individuals visiting the KwaPhuza border market (Fig. 1c), situated along the border between uMhlabuyalingana municipality, KZN and Maputo Province, Mozambique, for malaria and determining their recent and typical travel history. The third component was an entomological survey of potential vector populations within the study area in order to assess malaria risk and receptivity.

### Sampling frame

The community-based, household-level survey was restricted to two types of localities: those reporting at least one locally-acquired case (defined as a malaria infection acquired within that community as no travel to another malaria endemic region in past 14 days was reported) during the previous two malaria seasons, and those where MMPs are thought to frequent. As the edges of these communities are somewhat diffuse, the official administrative boundaries from Statistics South Africa (StatsSA) may potentially exclude portions of communities, so each StatsSA boundary was extended outwards by 1 km. These extended borders defined the sample frame and study localities. To obtain the study sample, study localities were subdivided into a grid comprised of 500 × 500-m blocks (Fig. 1c), and blocks were chosen at random (but with a probability proportion to the number of households therein). Inhabited blocks were randomly selected until 1351 households were identified, with the caveat that more blocks were selected from within uMhlabuyalingana municipality, given its larger size, and the large number of localities that the MMPs were thought to frequent. Assuming every house in the block would be sampled, an average of 3.7 individuals per household (as calculated by the National Census) and an anticipated refusal or absence rate of approximately 20%, sampling this number of households was designed to provide us with 4000 study participants. Based on the study sample size calculation, if no individual was found to be malaria positive, this number of participants would be sufficient evidence to show a prevalence of less than 0.15%.

At the KwaPhuza border market, a convenience sampling strategy was employed as the number of individuals using the border crossing is unknown and therefore unpredictable. All individuals entering the South African side of the border market on the day of sampling (every Wednesday during the 6-week survey period) were invited to participate. All those who consented were surveyed.

Entomological investigations were conducted in and within a 2 km radius of any surveyed household where a malaria case was detected during the survey. In study localities where no cases were detected, at least two randomly selected surveyed households were subjected to entomological investigations.

### Sample and field data collection

#### Blood and participant information collection

All individuals over 2 years of age present at the selected household or visiting KwaPhuza border market were invited to participate in the study. Prior to blood sampling and survey administration, written consent was obtained from individuals ≥ 18 years of age, with written consent from a guardian/caregiver/parent of individuals between 2 and 18 years. Assent was also obtained from children aged between 6 and 18 years.

All consenting participants from the community and border market were tested for malaria by standard RDT (First Response™ Malaria Ag *P. falciparum* HRP2 Detection Rapid Card Test, Premier Medical Corporation Ltd, India) and highly sensitive RDT (Alere™ Malaria AG P.F. Ultra Sensitive, Abbott, USA). This was done to compare the performance of the highly sensitive RDT to the standard RDT in a low-transmission rural South African setting. Filter-paper finger-prick blood samples were collected on Munktell TFN cards (Munktell, Germany) and labelled with unique patient identifiers to ensure linkage of molecular and demographic data. Air-dried blood samples were packaged individually in zip-lock packets containing desiccant and transported to the National Institute for Communicable Diseases (NICD) in Johannesburg for further analysis. Malaria RDT-positive individuals were treated on-site with artemether–lumefantrine in accordance with South African malaria treatment guidelines [14].

A paperless KAP survey designed to efficiently gather information deemed critical by the KZN Malaria Control Programme was administered to all consenting participants. Participants over the age of six were asked general knowledge questions while those 18 years and older were asked more in-depth attitude and practice questions. Detailed travel histories from all consenting individuals 18 years and older, visiting the border market, were obtained using a semi-structured paper-based questionnaire.

#### Entomological activities

At least two randomly surveyed households in each locality were visually inspected for indoor-resting adult mosquitoes. Households that received IRS during the current malaria season were randomly assessed for insecticide residual efficacy using the standard WHO cone bioassays [16] against an insecticide-sensitive *An. arabiensis* laboratory strain. Final mortality counts were taken 24 h post-exposure. Description and coordinates of any potential breeding site within a 2 km radius of a survey household identified during the entomological survey were recorded. Larvae, if present, were collected and transported to the malaria programme insectary in Jozini for further analysis.

### Laboratory analyses

#### Malaria asexual and sexual parasite detection

A modification of the pooling PCR method described by Hsiang et al. [17] was employed to confirm malaria infection and parasite species identification. Briefly, DNA extracted from master pools containing samples from five participants (two 6 mm in diameter samples per participant) using the QIAamp DNA mini extraction kit (Qiagen, Germany) was subjected to a cytochrome *b* nested PCR. Samples from any positive master pool were tested individually, followed by AluI enzyme restriction digestion for species determination. All falciparum-positive samples were assessed for the markers associated with artemisinin resistance [18] and lumefantrine tolerance [19, 20] as well as genotyped using 26 neutral microsatellite markers [21]. All collected blood samples, irrespective of malaria status, were also assessed for gametocyte carriage using the reverse transcriptase PCR method of Mlambo et al. [22].

#### Vector species identification and susceptibility testing

Larvae, reared to adulthood in the Jozini insectary, were morphologically identified using the keys of Gillies and Meillion [23] and Gillies and Coetzee [24], with sub-sets of the adults subjected to insecticide susceptibility testing using WHO susceptibility test kits [25]. Final mortality counts were taken 24 h post-exposure. Species identity was confirmed where necessary using the PCR methods of Koekemoer et al. [26] and Scott et al. [27] for *An. funestus* group and *An. gambiae* complex samples, respectively.

### Malaria case classification

Local case: a malaria case within a malaria receptive area where local transmission cannot be disproved and there is no recent history of travel to another malaria endemic area.

Imported case: a malaria case whose source of infection can be trace to an area outside of South Africa where the patient has recently travelled.

### Statistical and geospatial analyses

Data cleaning and analyses were conducted using Tableau Prep and Desktop (Tableau Software, Seattle, WA, USA), RStudio (RStudio, Vienna, Austria) and Stata 15.0 (Stata Corp, College Station, Texas, USA). Odds ratios (OR) associated with malaria risk were generated using univariate analysis and variable logistic regression models which took into account correlations at the locality level. Confidence limits were set at 95% with p < 0.05 considered significant. Geospatial mapping and analysis was conducted using ArcGIS (Esri, Redlands, California. USA).

## Results

Initially 2096 community members from 1214 households consented to malaria testing and KAP survey administration. However, two participants withdrew their consent after receiving their test results, resulting a final study sample of 2094 participants from 1214 households. Willingness to participate in the survey was extremely high, with only one household head denying the survey team permission to administer the survey in their household. Data on the number of households where no one was present at the time of the survey were unfortunately not collected. Anecdotal evidence however suggests that this number was low. At the border-market, all 885 individuals approached, consented to malaria testing, and provided both travel histories and a filter-paper blood sample. The targeted sample size of 4000 was not achieved due to fewer participants than expected present at the selected households at the time of survey administration together with a week-long disruption of survey activities due to localized flooding.

**Fig. 2:**
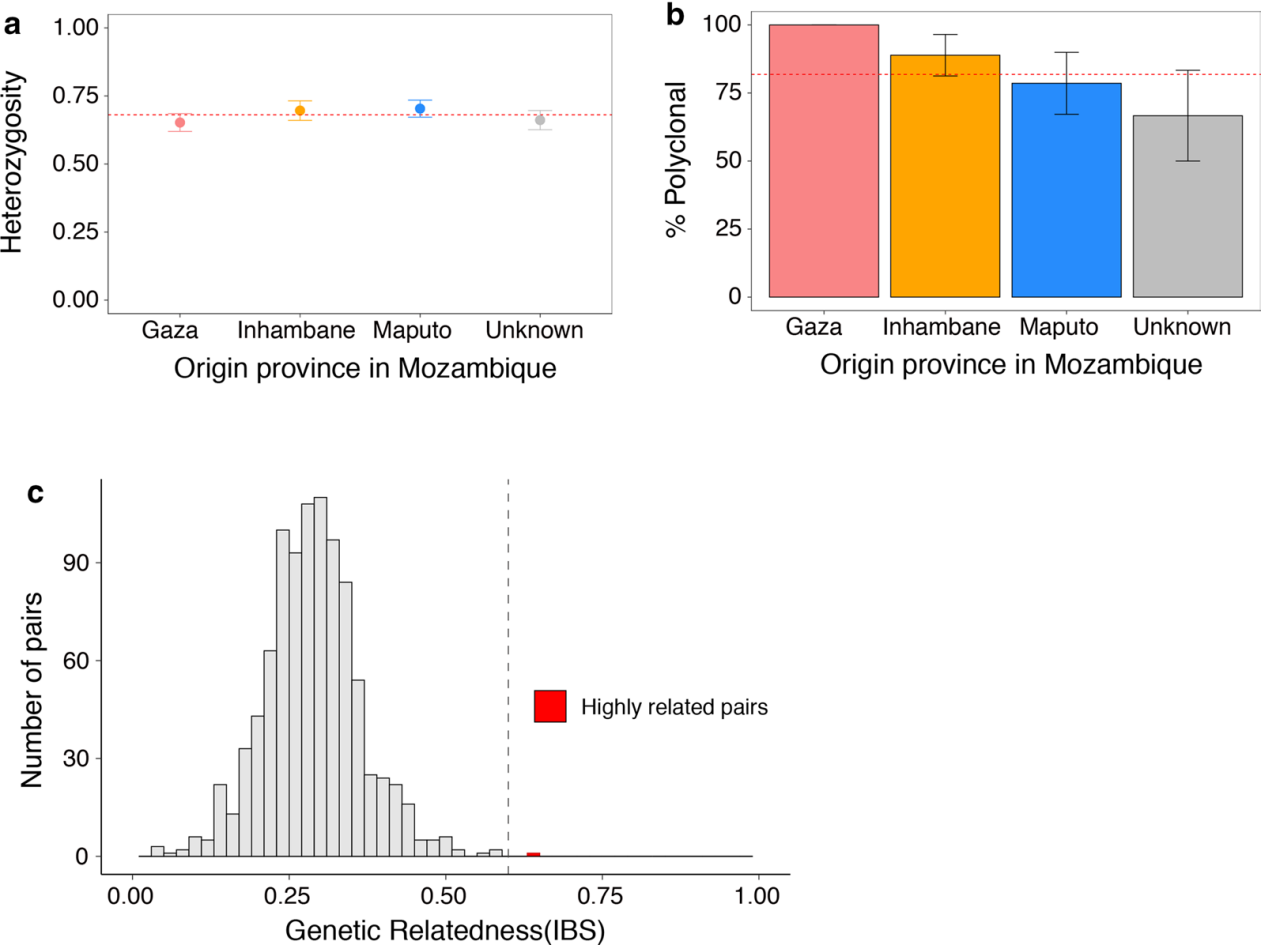
Within-host and population diversity of malaria parasite isolates collected during the prevalence survey, **a** population level genetic diversity measured as the distribution of heterozygosity in 26 microsatellites. The dashed red line indicates the mean heterozygosity = 0.68. Values for H_e_ range from 0–1, with 0 representing no diversity and 1 representing 100% of alleles being different. **b** Complexity of the infections as measured by number of clones present by source locality with Mozambique and **c** pairwise genetic relatedness between samples, calculated using identity by state (IBS) metric including all alleles detected in polyclonal samples. A single highly related pair (genetic relatedness > 0.6) was identified and is shown in red

Malaria prevalence was low, with only 67 (2%) of the 2979 sampled individuals testing positive for malaria by the standard falciparum-specific malaria RDT. The malaria burden however differed significantly between the community (2/2094) and border market (65/885; p < 0.0001), with the border market accounting for 97% (65/67) of all cases detected. Based on travel history data, all cases detected during this study, both at the border market and in the community, were classified as imported from neighbouring Mozambique. Good quality genotyping data were available for 46 parasite isolates (68.7%) and revealed that the parasite isolates were genetically diverse and complex with limited genetic relatedness (Fig. 2), suggesting frequent and random mixing of parasites, consistent with the characteristics of imported infections from high transmission areas. Pairwise genetic relatedness assessment identified a single pair of highly related infections (identity by state (IBS) > 0.6, Fig. 2c), suggesting limited direct transmission between these Mozambican individuals. Although case notification data confirmed that these infections were detected on the same day at the KwaPhuza border-market, information on source location within Mozambique was not collected. No additional cases were detected by ultra-sensitive RDT or pooled PCR, with gametocyte carriage limited to the 67 individuals found to be malaria positive. All parasite isolates carried the lumefantrine-tolerance marker but none of the validated artemisinin resistance markers.

Within the community, adult females were over-represented in the sample (70.5%, p < 0.0001). The unemployment rate was incredibly high (80%, p < 0.0001) in spite of just over 50% (1031/2001) of the sampled population having secondary education or higher (Table 1). Previous exposure to malaria was extremely low, with only 1.6% (32/1998) of the sampled individuals reporting having had malaria. Despite the limited exposure to malaria, the majority of the survey participants (94%), had heard of malaria (OR 2.70; CI 2.52–2.88; p < 0.0001), and knew that if left untreated malaria could be fatal (OR 4.38; CI 3.97–4.78; p < 0.0001). Headaches (86%) and having chills or feeling cold (77%) were the two symptoms most frequently associated with malaria. Surprisingly, only 61.3% of the individuals interviewed associated fever with malaria. Although over 90% of all homesteads visited had been exposed to IRS operations during the current malaria season (OR 2.39; CI 2.23–2.54; p < 0.0001), localities within Jozini municipality were more likely to be sprayed than those in uMhlabuyalingana municipality (OR 1.57; CI 1.07–02.32; p = 0.022). Bed net ownership was very limited with only 4% (85/2090) of the individuals interviewed reporting owning a bed net. Use of a bed net differed markedly between the study municipalities, with usage significantly higher in the rural municipality of uMhlabuyalingana (53.2%) compared to peri-urban Jozini (39.1%; Table 1). Additional survey data can be found in Additional file 1: Table S1.

**Table 1:** Demographic characteristics and risk factors associated with *Plasmodium falciparum* malaria from the community-based KAP survey by study municipality in uMkhanyakude district, KwaZulu-Natal

Risk factor	Jozini N (%)	p value	UMhlabuyalingana N (%)	p value	Total N (%)	p value
Gender						
Female	368 (64.7)	< 0.0001	1109 (72.7)	< 0.0001	1477 (70.5)	< 0.0001
Male	201 (35.3)		416 (27.3)		617 (29.5)	
Age (years)						
Under 5	43 (7.4)	–	35 (2.3)	–	77 (3.7)	–
5–20	71 (12.5)	< 0.0001	141 (9.3)	< 0.0001	212 (10.1)	< 0.0001
21–60	386 (67.8)	< 0.0001	1084 (71.1)	< 0.0001	1470 (70.2)	< 0.0001
Over 60	70 (12.3)	< 0.0001	265 (17.4)	< 0.0001	335 (16)	< 0.0001
Education						
No education	131 (25.2)	–	403 (27.2)	–	534 (26.7)	–
Primary education	106 (20.4)	< 0.0001	330 ( 22.3)	< 0.0001	436 (21.8)	< 0.0001
Secondary education and above	282 (54.3)	< 0.0001	749 (50.5)	<0.0001	1031 (51.5)	< 0.0001
Employed						
Yes	172 (30.1)	< 0.0001	282 (18.5)	< 0.0001	454 (21.7)	< 0.0001
No	399 (69.9)		1241 (81.5)		1640 (78.3)	
Previously had malaria						
Yes	7 (1.4)	< 0.0001	25 (1.7)	< 0.0001	32 (1.6)	< 0.0001
No	509 (98.6)		1457 (98.3)		1966 (98.4)	
IRS in the past 6 months						
Yes	524 (93.9)	< 0.0001	1 383 (90.8)	< 0.0001	1907 (91.6)	< 0.0001
No	34 (6.1)		141 (9.2)		175 (8.4)	
Bed net ownership						
Yes	23 (4)	< 0.0001	62 (4)	< 0.0001	85 (4)	< 0.0001
No	546 (96)		1459 (96)		2005 (96)	
Bed net use						
Yes	9 (39.1)	< 0.0001	33 (53.2)	< 0.0001	42 (49.4)	< 0.0001
No	14 (60.9)		29 (46.8)		43 (50.6)	
Recent travel out of KZN						
Yes	20 (3.9)	< 0.0001	62 (4.2)	< 0.0001	82 (4.1)	< 0.0001
No	497 (96.1)		1421 (95.8)		1918 (95.9)	

**Fig. 3:**
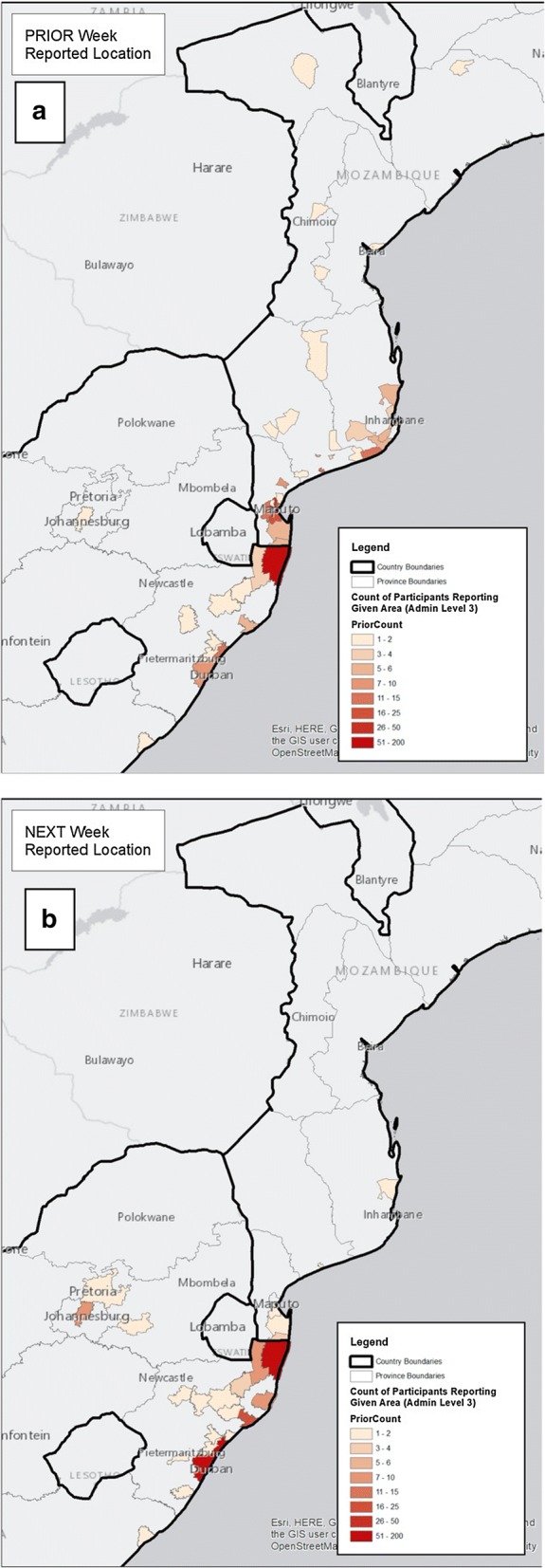
**a** Localities the individuals sampled at KwaPhuza Border Market arrived from and **b** localities to which they were transiting

In contrast to the community-based survey, similar numbers of males and females were sampled at the KwaPhuza border market (p = 0.591). Males tested at the border market were more likely to be infected with malaria compared to the females (OR 1.98; CI 1.26–2.70; p < 0.0001). The majority of the individuals who provided travel information indicated that they were planning on travelling to major South African cities (Fig. 3).

**Fig. 4:**
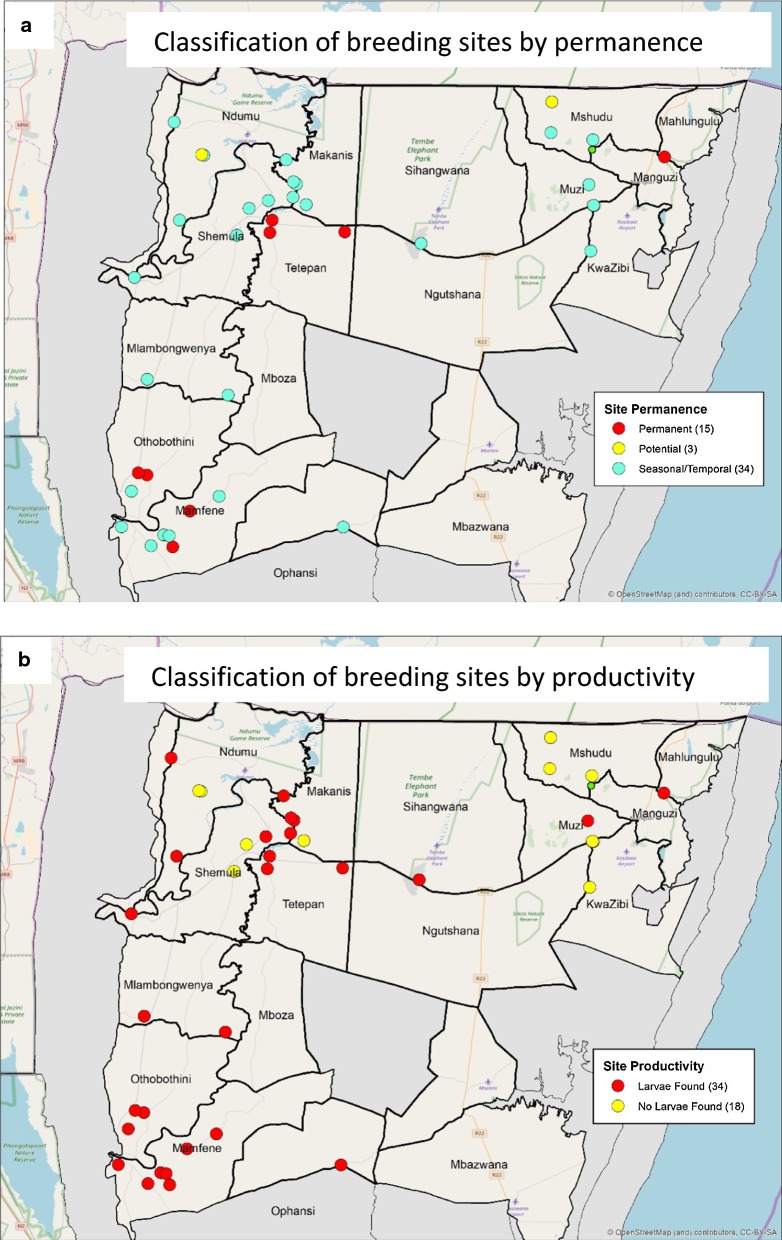
Breeding sites identified during the prevalence survey classified by **a** site permanence and **b** productivity

All potential *Anopheles* breeding sites identified during the survey were geolocated, classified according to breeding status and mapped (Fig. 4). While no adult mosquitoes were found at any of the survey sites, larvae belonging to the *An. gambiae* complex and *An. funestus* group were collected from a number of potential sites across the study area. Molecular analysis confirmed the majority to be *An. arabiensis* (Table 2), with members of the *An. funestus* group extremely rare. Although all vector species identified in the study were found to be susceptible to DDT, populations of *An. arabiensis* and *Anopheles pretoriensis* with reduced pyrethroid-susceptibility were detected (Table 3). Quality of IRS operations varied between the different localities (Table 4).

**Table 2:** PCR-based species identification of a subset of *Anopheles* larvae collected from 10 potential breeding sites within the study localities in uMkhanyakude district, KwaZulu-Natal, during the malaria prevalence survey in 2018

Complex/group	Total N	Species	Total N (%)
*An. gambiae* complex	80	*An. arabiensis*	56 (70)
		*An. quadriannulatus*	21 (26)
		Not identified	3 (4)
*An. funestus* group	2	*An. parensis*	1 (50)
		Not identified	1 (50)

**Table 3:** Insecticide susceptibility status of *Anopheles* mosquito samples^a^ collected from potential breeding sites within uMkhanyakude district, KwaZulu-Natal, during the prevalence survey in 2018

Species	Insecticide	Number of mosquitoes tested	% mortality 24 h post exposure	Susceptibility status
*An. gambiae* complex^b^	4% DDT	20	100	Susceptible
*An. pretoriensis*	4% DDT	11	100	Susceptible
*An. arabiensis*	0.05% Deltamethrin	5	80	Resistant
*An. pretoriensis*	0.05% Deltamethrin	7	85.7	Resistant

^a^ Larvae of mixed gender were reared to adulthood (2–3 days old) in the Jozini insectary and subjected to the standard WHO insecticide susceptibility testing assay

^b^Species identification was not confirmed by PCR

**Table 4:** Assessment of insecticide residual efficacy in selected households from study localities that had IRS in the past 6 months using cone bioassays with insecticide-sensitive laboratory-bred *Anopheles arabiensis* mosquitoes

Municipality	Locality	Date of last IRS round	Surface type	Insecticide used	% Mortality after 24 h^a^		
					Top^b^	Middle^b^	Bottom^b^
UMhlabuyalingana	Makanis	2017 Oct 19	Cement	DDT	100	100	60
uMhlabuyalingana	Makanis	2017 Oct 19	Painted	DDT	100	100	100
uMhlabuyalingana	Manguzi	2017 Oct 19	Painted	Deltamethrin	100	90	90
uMhlabuyalingana	Manguzi	2017 Oct 19	Painted	Deltamethrin	100	80	100
uMhlabuyalingana	Manguzi	2017 Oct 18	Painted	Deltamethrin	100	100	100
uMhlabuyalingana	Mshudu	2017 Nov 1	Cement	DDT	100	100	90
uMhlabuyalingana	Muzi	2017 Dec 5	Painted	Deltamethrin	100	70	100
uMhlabuyalingana	Muzi	2017 Dec 5	Cement	DDT	100	100	80
uMhlabuyalingana	Muzi	2018 Jan 15	Painted	DDT	100	100	100
uMhlabuyalingana	Muzi	2018 Jan 15	Painted	Deltamethrin	100	100	100
uMhlabuyalingana	Muzi	2018 Jan 15	Painted	Deltamethrin	100	100	90
Jozini	Ndumo	2017 Oct 4	Mud	DDT	100	100	100
Jozini	Ndumo	2017 Oct 4	Cement	DDT	100	100	100
Jozini	Ndumo	2017 Oct 4	Painted	Deltamethrin	100	100	100
Jozini	Ndumo	2017 Oct 4	Cement	DDT	100	100	100
Jozini	Ndumo	2017 Nov 14	Painted	DDT	93	100	94
Jozini	Ndumo	2017 Nov 14	Cement	DDT	100	100	100
Jozini	Ndumo	2017 Dec 20	Painted	Deltamethrin	100	100	100
Jozini	Ndumo	2017 Dec 20	Plastered	DDT	100	100	92
Jozini	Ndumo	2017 Dec 21	Painted	Deltamethrin	100	100	85
Jozini	Ndumo	2017 Dec 20	Cement	DDT	100	100	100
uMhlabuyalingana	Ngutshana	2017 Jan 20	Painted	Deltamethrin	60	50	90
Jozini	Shemula	2018 Jan 30	Painted	Deltamethrin	100	100	100
uMhlabuyalingana	Sihangwani	2017 Sep 20	Plastered	DDT	60	40	90
uMhlabuyalingana	Sihangwani	2017 Sep 20	Painted	Deltamethrin	100	70	60
uMhlabuyalingana	Sihangwani	2017 Sep 20	Plastered	DDT	100	90	100
uMhlabuyalingana	Tetepan	2017 Nov 3	Cement	Deltamethrin	100	100	100
uMhlabuyalingana	Tetepan	2017 Nov 3	Cement	Deltamethrin	90	100	100
uMhlabuyalingana	Tetepan	2017 Nov 3	Cement	Deltamethrin	100	100	100
uMhlabuyalingana	Tetepan	2017 Nov 3	Cement	Deltamethrin	100	100	100

^a^A control was included in each experiment, with 100% survival reported

^b^Cones were place at three points on the wall surface of each selected homestead, the top, middle and bottom, to assess spray-quality across the entire wall surface

## Discussion

Achieving WHO-verified malaria elimination status in all three endemic districts in KZN by 2021 is a core objective of South Africa’s current malaria elimination strategy. This study provided additional evidence of marked progress towards this goal in KZN, by confirming limited indigenous (autochthonous) malaria transmission in the province. However, vigilance in malaria surveillance and response must remain a priority, as the attainment of malaria elimination is under threat from numerous factors, including malaria importation from neighbouring high-prevalence countries and insecticide resistance.

The study parasite isolates were found to be highly diverse and complex with limited levels of genetic relatedness, distinctive features of parasites from a high transmission area. These findings supported the classification of all detected cases (97% of which were detected during the border market survey at the informal KwaPhuza border market) as imported based on travel history data. The rapid development of both formal and informal global transport networks has increased human mobility and the speed infectious diseases spread [28]. Within southern Africa where malaria distribution is extremely heterogeneous and country borders porous, human population movement (HPM), predominately by road [28], links areas of differing malaria receptivity. Areas of low receptivity, where malaria transmission would not normally be sustained, can experience persistent transmission [29] due to a continually replenished parasite pool [30]. This constant introduction of parasites into receptive areas by HPM has been identified as a major contributory factor in the failure of previous elimination campaigns [31].

All the identified malaria carriers in this study were asymptomatic, a likely consequence of acquired immunity due to Mozambique’s higher transmission intensity. The majority of these malaria carriers would have most likely evaded passive case detection, potentially seeding and sustaining secondary (introduced) transmission in receptive areas of KZN, had they not been detected and treated at the informal border crossing. In addition, many of MMPs interviewed indicated that they were transiting to major non-endemic cities within South Africa, potentially placing an increased burden on the health systems in these cities, if they became symptomatic and required treatment. As this survey was conducted outside of the peak transmission periods, it is very likely that levels of both HPM and asymptomatic carriage have been significantly underestimated, notwithstanding the likelihood that population movements differs across the year. This, together with the study findings strongly emphasize the critical role the mobile border malaria surveillance units play in reducing the opportunities for these asymptomatic carriers to seed secondary transmission in KZN, particularly as entomological investigations confirmed the presence of numerous receptive areas within the province. The ability of the mobile surveillance units to prevent onward transmission has been further enhanced with the recent addition of the gametocidal drug, primaquine, to their case management toolkit.

Acknowledging the threat asymptomatic malaria importation poses to malaria elimination in southern Africa, the MOSASWA (Mozambique, South Africa, Swaziland/Eswatini) cross-border collaboration has implemented two strategies to address asymptomatic malaria importation both in source and sink areas [32]. The first aims to reduce transmission in southern Mozambique by strengthening human capacity to effectively malaria control and improving intervention coverage in Maputo, Gaza and Inhanbane Provinces while the second focusses on detecting and treating malaria infections before they reach receptive areas within KZN. However, more detailed intelligence on the demographics and movements of the MMPs is required to inform targeted active case detection activities. Once this information is sourced, the appropriate effective active case detection activities must be expanded and concentrated at known border crossings (formal and informal) and gathering points of MMPs across South Africa, to facilitate the prompt detection of malaria and improved malaria awareness.

The current point of care diagnostic in South Africa, a conventional falciparum-specific RDT, appeared to be sensitive enough to detect asymptomatic carriage as no additional malaria cases were detected by standard PCR or ultra-sensitive falciparum-specific RDTs. While similar results have been observed in low-transmission areas of the Gambia [33], they differ from a study in Ethiopia [34] which found the same ultra-sensitive RDT used in this study to be more sensitive compared to conventional RDTs when detecting asymptomatic malaria. A possible reason for this difference could be sensitivity differences between the conventional RDTs used in both studies. It has been suggested that low-density infections, including submicroscopic infection, are very prevalent in very low-transmission settings [34, 35] and are capable of sustaining transmission [36]. Detection of these infections however depends on the molecular methods used and more critically sample volume [37]. Unfortunately, the collection and appropriate storage of high-volume blood samples required for these ultrasensitive techniques was not logistically feasible during the survey. Investigations into the prevalence of low-density infections and their contribution to sustained transmission in KZN should be considered.

The severe under-sampling of school-going children and employed adults, a consequence of the community-based survey being conducted on weekdays between the hours of 09h00 and 15h00 when these populations groups were not present, was a major limitation of this study. As these groups are often highly mobile, their exclusion could have biased the finding of very limited indigenous transmission within the community. However, given the limited number of indigenous cases reported from the communities within the study area during the previous two malaria seasons, this seems highly unlikely.

Encouragingly, despite KZN’s current extremely low malaria prevalence, awareness of malaria and its associated dangers was high within the community. It was however surprising to note that while most participants knew the common signs and symptoms of malaria, headaches and feeling cold were more frequently listed than fever. A similar trend was observed in an earlier study [38], highlighting the need for targeted health promotion around fever and malaria, particularly as fever is used as a prescreening tool for malaria testing at all South African public health facilities.

Sustained effective coverage of efficacious vector control inventions is fundamental to the success of any elimination strategy [39]. The suboptimal IRS coverage driven by an increase in western-style homesteads and substandard spray quality detected in certain localities are potential obstacles to malaria elimination in KZN. In areas where IRS operations are still feasible, they must be closely monitored and evaluated, ideally in real time, to ensure the 90% coverage target of good quality IRS, is achieved. Additionally other vector control interventions such as improving housing structures and/or larval source management should be considered. Currently in KZN, larval source management is employed in an ad hoc uncoordinated manner. The KZN control programme should consider upscaling its use; guided by regularly updated breeding site maps and comprehensive data management, to control out-door resting vector populations associated with sustaining residual transmission in KZN [15, 40]. As vector populations with reduced susceptibility to pyrethroids were detected in this study and have been confirmed in neighbouring Mozambique [41], it is imperative that resistance susceptibility testing becomes a routine programmatic activity, with the data generated used to inform insecticide selection.

## Conclusion

This study identified the continual introduction of malaria parasites into receptive areas through asymptomatic importation from neighbouring Mozambique as a potential significant driver of residual indigenous transmission in northern KZN. Routine active detection at known informal border crossings, the introduction of the transmission-blocking drug primaquine and the strengthening of cross-borders initiatives are a few of the elimination strategies implemented to address imported and introduced malaria. It should be noted that current research suggests that if these interventions are to have any impact on suppressing indigenous transmission, a coverage of at least 80% must be achieved and maintained for a significant length of time. In addition, the receptivity and vulnerability of many localities within KZN was confirmed, highlighting the need for sustained, optimum coverage of a package of targeted effective vector control interventions.

## Supplementary information

**Additional file 1: Table S1.** Additional risk factors associated with *Plasmodium falciparum* malaria from the community-based KAP survey by study municipality in uMkhanyakude district, KwaZulu-Natal.

## Data Availability

The datasets used and/or analysed during this study are available from the corresponding author on reasonable request.

## Abbreviations

DDT: Dichlorodiphenyltrichloroethane
IRS: Indoor residual spraying
KAP: Knowledge, attitudes and practices
KZN: KwaZulu-Natal
NICD: National Institute for Communicable Diseases
MMPs: Migrant and mobile populations
MOSASWA: Mozambique, South Africa, Swaziland/Eswatini
PCR: Polymerase chain reaction
RDT: Rapid diagnostic test
