# Supplementary material for: High levels of imported asymptomatic malaria but limited local transmission in KwaZulu-Natal, a South African malaria-endemic province nearing malaria elimination
Source: Malar J. 2020 Apr 15;19:152. doi: 10.1186/s12936-020-03227-3 (PMC7161075; doi:10.1186/s12936-020-03227-3)
Supplement: Supplementary file 1 — Additional file 1: Table S1. Additional risk factors associated with Plasmodium falciparum malaria from the community-based KAP survey by study municipality in uMkhanyakude district, KwaZulu-Natal. [file 12936_2020_3227_MOESM1_ESM.docx]

Table S1. Additional risk factors associated with *Plasmodium falciparum* malaria from the community-based KAP survey by study municipality in uMkhanyakude district, KwaZulu-Natal

| Risk Factor | Jozini  N (%) | uMhlabuyalingana  N (%) | Total  N (%) |
| --- | --- | --- | --- |
| **Household Structure** |  |  |  |
| Traditional  Mixed Material  Brick/Cement  **Reason for no IRS**  Away from home  Sick person in the house  Furniture could not be moved  Never offered  **Travel outside KZN**  Mozambique  South Africa  Eswatini  Malawi  **Fever in the past month**  Yes  No  **Place treatment sought**  Government facility  Private facility  Traditional healer  **Days after fever sought treatment**  1 day  3 days  5 days  1 week or longer  Do not remember  **Personal protection used**  Bednets  Chemoprophylaxis  Coils  Fire/Smoke  Repellents | 13 (4.7)  149 (54.4)  112 (40.9)  4 (23.5)  0  1 (5.9)  12 (70.6)  4 (20)  13 (65)  2 (10)  1 (5)  4 (1)  515 (99)  3(75)  1 (25)  0  1 (25)  0  0  2 (50)  1 (25)  9 (81.8  1 (9.1)  1 (9.1)  0  0 | 40 (4.4)  452 (49.5)  448 (46.1)  87 (65.9)  9 (6.8)  2 (1.5)  34 (25.8)  47 (75.8)  15 (24.2)  0  0  14 (1)  1 468 (99)  14 (100)  0  0  1 (7.1)  2 (14.3)  1 (7.1)  0  10 (71.4)  33 (71.7)  2 (4.3)  2 (4.3)  1 (2.2)  8 (17.4) | 53 (4.4)  601 (49.5)  560 (46.1)  91 (61.1)  9 (6)  3 (2)  46 (30.9)  51 (62.2)  28 (34.2)  2 (2.4)  1 (1.2)  18 (1)  1 983 (99)  17 (94.4)  1 (5.6)  0  2 (11.1)  2 (11.1)  1 (5.6)  2 (11.1)  11 (61.1)  42 (73.7)  3 (5.3)  3 (5.3)  1 (1.8)  8 (14) |
